# The effect on women’s health of extending parental leave: a quasi-experimental registry-based cohort study

**DOI:** 10.1093/ije/dyac198

**Published:** 2022-10-14

**Authors:** Emilie Courtin, Andreas Rieckmann, Jessica Bengtsson, Vahe Nafilyan, Maria Melchior, Lisa Berkman, Naja Hulvej Rod

**Affiliations:** Department of Public Health, Environments and Society, London School of Hygiene and Tropical Medicine, London, UK; Department of Public Health, Section of Epidemiology, University of Copenhagen, Copenhagen, Denmark; Department of Public Health, Section of Epidemiology, University of Copenhagen, Copenhagen, Denmark; Department of Public Health, Environments and Society, London School of Hygiene and Tropical Medicine, London, UK; King’s Business School, King’s College London, London, UK; Institut Pierre Louis d'Epidémiologie et de Santé Publique, INSERM UMR S 1136, Sorbonne University, Paris, France; Harvard Center for Population and Development Studies, Harvard University, Cambridge, MA, USA; Department of Public Health, Section of Epidemiology, University of Copenhagen, Copenhagen, Denmark

**Keywords:** Parental leave, mental disorders, quasi-experiment, social determinants of health, women’s health

## Abstract

**Background:**

Parental leave policies have been hypothesized to benefit mothers’ mental health. We assessed the impact of a 6-week extension of parental leave in Denmark on maternal mental health.

**Methods:**

We linked individual-level data from Danish national registries on maternal sociodemographic characteristics and psychiatric diagnoses. A regression discontinuity design was applied to study the increase in parental leave duration after 26 March 1984. We included women who had given birth between 1 January 1981 and 31 December 1987. Our outcome was a first psychiatric diagnosis following the child’s birth, ascertained as the first day of inpatient hospital admission for any psychiatric disorder. We presented cumulative incidences for the 30-year follow-up period and reported absolute risk differences between women eligible for the reform vs not, in 5-year intervals.

**Results:**

In all, 291 152 women were followed up until 2017, death, emigration or date of first psychiatric diagnosis. The median follow-up time was 29.99 years, corresponding to 10 277 547 person-years at risk. The cumulative incidence of psychiatric diagnoses at 30 years of follow-up was 59.5 (95% CI: 57.4 to 61.6) per 1000 women in the ineligible group and 57.5 (95% CI: 55.6 to 59.4) in the eligible group. Eligible women took on average 32.85 additional days of parental leave (95% CI: 29.20 to 36.49) and had a lower probability of having a psychiatric diagnosis within 5 years [risk difference (RD): 2.4 fewer diagnoses per 1000 women, 95% CI: 1.5 to 3.2] and up to 20 years after the birth (RD: 2.3, 95% CI: 0.4 to 4.2). In subgroup analyses, the risk reduction was concentrated among low-educated, low-income and single women.

**Conclusions:**

Longer parental leave may confer mental health benefits to women, in particular to those from disadvantaged backgrounds.

Key Messages

- We linked individual-level data from Danish national registries and used a quasi-experimental approach to investigate the effects of a generous expansion of maternity leave duration on maternal mental health up to 30 years after the reform.
- Eligibility to longer parental leave was associated with reductions in the risk of receiving a psychiatric diagnosis for up to 20 years after the policy introduction.
- Women with low educational levels, below average income and/or without a partner at time of birth seemed to benefit the most from the reform.
- Increasing the length of parental leave may have a beneficial impact on women’s mental health, in particular for socially disadvantaged mothers.

## Introduction

New mothers are at increased risk of a range of psychiatric disorders in the postpartum period,^1^^,^^2^ with lasting consequences on family health^3^^,^^4^ and on the risk of developing subsequent major depression and other disorders later in life.^5^ Parental leave, which allows parents to take time off work to care for newborn children, might reduce conflicting work and family demands, allow mothers to bond with their child and reduce their risk of psychiatric disorder in the short and longer term.

Evidence to date on the impact of parental leave policies on mothers’ mental health has, however, several limitations.^6^^,^^7^ First, the bulk of the evidence has focused on children’s outcomes, with small positive effects on birthweight and reductions in infant mortality rates.^8^ Second, studies documenting an association between leave duration and improved mental health have generally adopted cross-sectional designs, which are vulnerable to confounding bias and reverse causality.^9–14^ Although recent studies have exploited policy variations within or across countries to overcome this issue, they report mixed findings, with positive effects of the introduction or extension of paid leave on breastfeeding,^15^^,^^16^ parental physical^17^^,^^18^ and mental health^17–20^ and alcohol use;^17^ but no impact on postpartum depression^21^^,^^22^ or maternal self-reported health.^22^ Third, there is a paucity of studies evaluating long-term effects of parental leave. Only one study found that women who benefited from more generous maternal leave policies reported lower levels of depressive symptoms in old age.^19^

The life course impact of parental leave policies on women’s mental health warrants further research, in particular using a robust quasi-experimental approach in a large sample of women with no loss to follow-up. Under a number of conditions, this approach is less prone to confounding at the individual level because the allocation to the policy is expected to be unrelated to other factors that cause the outcome of interest.^23^ Which subgroups of women are more likely to benefit from parental leave policies also remains an open question.

This paper investigates the impact of a 6-week expansion of parental leave on women’s mental health. First, we applied a quasi-experimental approach to estimate the effect of a Danish reform which significantly extended parental leave on objective mental health outcomes in over 290 000 women. Second, we exploited rich registry data on maternal characteristics and health histories to investigate which sub-groups of women benefited the most from the policy. Finally, we used the full 30 years of follow-up data available to identify short-term as well as lasting effects of the policy on maternal mental health.

## Methods

### Study setting

In July 1984, Denmark extended the duration of paid parental leave from 14 to 20 weeks. The reform was universal, with a compensation rate of a maximum of DKK 2008 (about $201.6 in 1984 USD) per week, similar to pre-reform levels. The additional 6 weeks of leave could be shared between the mother and father but very few fathers took leave. The reform was implemented on 1 July 1984, but women who had already started their maternity leave less than 14 weeks before the reform were also eligible. The birth date cut-off for eligibility was consequently 26 March 1984 (Supplementary Appendix 1, available as Supplementary data at *IJE* online).^24^

### Data sources

**Figure 1:**
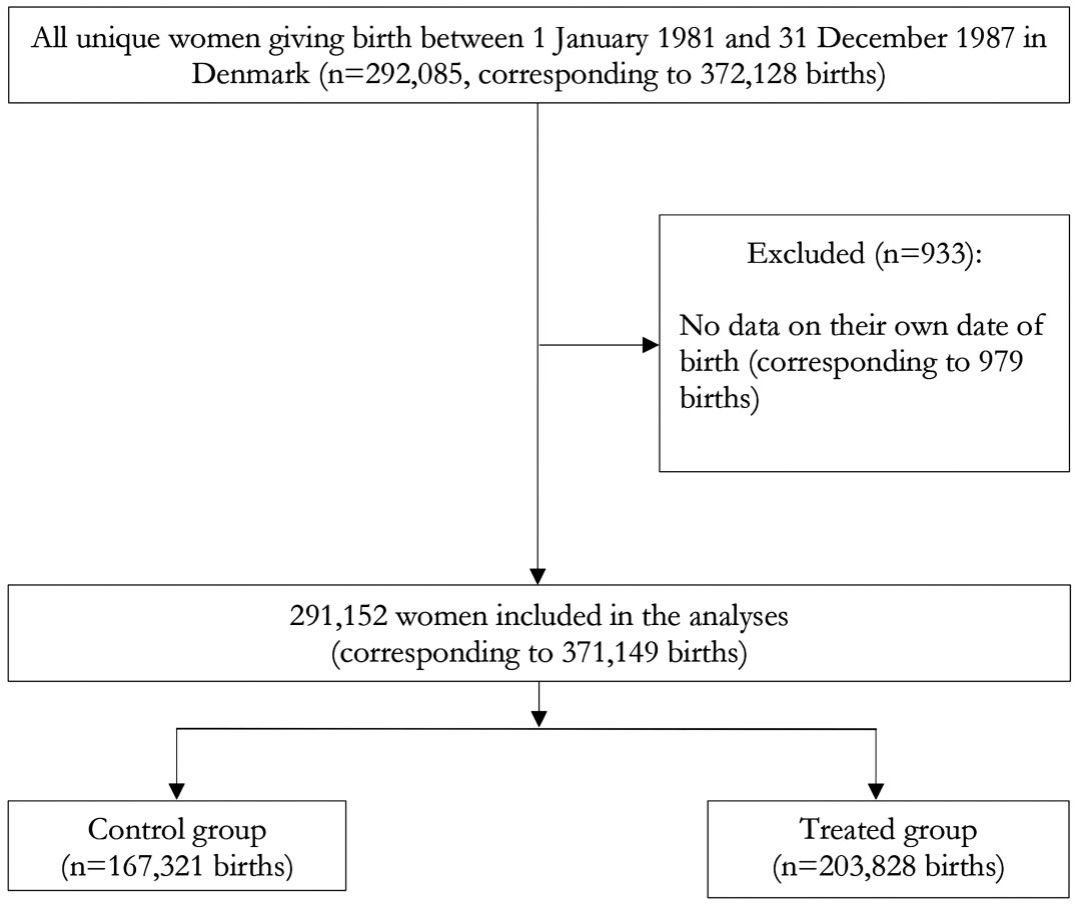
Study population. Women might have given birth in either or both control and treated groups during our time frame. We include robust standard errors clustered at the mother’s level to account for this issue in all models, and show that our findings are robust to a sensitivity analysis focusing on the firstborn child (see Supplementary Appendix 3, available as Supplementary data at *IJE* online for additional details about our model)

All live births and all residents in Denmark are assigned a unique identification number that allows linkages of individual-level data across Danish national registries. The Danish Civil Registration System^25^ includes the exact date of birth and allows a child to be linked to his/her parents. We identified all mothers who gave birth between 1 January 1981 and 31 December 1987 from the Danish Civil Registration System (*n* = 291 152). We excluded 933 women whose date of birth was not available (979 births). We included women who gave birth to twins or triplets as well as women who gave birth multiple times during our time frame. We included a total of 291 152 mothers with 371 149 births within our time frame (Figure 1).

Information on inpatient psychiatric diagnoses came from the Danish Psychiatric Central Research Register.^26^ Maternity leave duration was obtained from the Income Statistics Register.^27^ This information is available starting only in 1984, so our analysis of the impact of the reform on leave duration relies on a smaller sample defined as women giving birth close to the cut-off for the reform (1 February 1984 to 31 May 1984, *n *= 17 719). We considered four factors that might cause effect modification. Low birthweight (below 2500 g at birth vs over or equal to 2500 g) was used as an indicator of child vulnerability. Mother’s partnership status at the time of the child’s birth (partnered vs not partnered), family educational attainment (coded as under 10 years of education, 10 to 12 years and above 12 years, with the family assigned to the highest educational level registered for either parent) and income (above vs below the predicted average income) were used as indicators of the mother’s own social resources and potential vulnerability. These linkages were conducted as part of the DANLIFE cohort.^28^

### Outcomes

Our outcome of interest was first inpatient diagnosis of any psychiatric disorder (ICD-10 codes F00-F99, ICD-8 codes 290-319) in mothers. We used both primary and secondary diagnoses.

### Statistical analysis

We used the sharp increase in parental leave duration induced by the 1984 reform to conduct a regression discontinuity analysis.^29^^,^^30^ This approach tests whether the increase in leave duration was associated with a corresponding change in the risk of psychiatric disorders. We compared women who gave birth up to 3 years before the reform and were not eligible for the extended leave with women who gave birth up to 3 years after and could benefit from up to 6 additional weeks of leave. This approach mimics a randomized trial, as eligibility for the reform is solely based on the child’s date of birth and can be considered as good as random. We confirmed that there was no evidence of manipulation around the cut-off, i.e. no jump in the number of births at the threshold for eligibility for the reform (Supplementary Appendix 2, available as Supplementary data at *IJE* online).

The regression discontinuity design was combined with a time-to-event analysis to account for censored data. Each mother included in our analysis was followed until the date of first psychiatric diagnosis or censoring (31 December 2017, death, emigration), whichever came first. We defined the date of first diagnosis after giving birth as the first day of inpatient treatment that led to the assignment of a psychiatric diagnosis. We estimated a hazard function with the reform groups as strata and the years of follow-up since the birth as the underlying scale. We accounted for quadratic time trends by including a linear and squared variable of time since the reform in the model. Robust standard errors were clustered at the mother’s level to account for women giving birth multiple times in our time frame. Cumulative incidence functions (1-Kaplan–Meier survival plots) and associated 95% confidence intervals adjusted for the cohort effect were predicted for each stratum and absolute risk differences at 5-year intervals for the full duration of follow-up were estimated with 2.5% and 97.5% quantiles using 100 000 simulations. We interpreted our results in terms of probabilities rather than hazards, as we reported cumulative incidence. To investigate whether our effects were concentrated in specific subgroups, we stratified our analyses by child birthweight, partnership status, family education level and mother’s income. We conducted four sensitivity analyses^1^: we investigated whether the transition from ICD-8 to ICD-10 in 1994^26^ affected our results, by including this as a time-dependent variable^2^; we tested the effect of ‘placebo’ reforms on our outcome^3^; we assessed whether the bandwidth size affected our results^4^; and we assessed whether focusing only on the first child born influenced our results. Further details about the statistical analyses are provided in Supplementary Appendix 3, available as Supplementary data at *IJE* online.

## Results

We included 291 152 mothers who gave birth between 1 January 1981 and 31 December 1987. They were followed for 10 277 547 person-years (mean 27.7, median 30.0 years, maximum 30 years); 32 288 mother observations were lost to follow-up (18 072 due to emigration and 14 216 due to deaths). In our analytical sample, 167 321 births happened before the 26 March 1984 (control group, mean age of mothers 27.2) and 203 828 after that date (treated group, mean age of mothers 27.7). We recorded 16 460 psychiatric diagnoses during our follow-up period. Pre-reform characteristics were broadly similar across the two groups (Table 1).

**Table 1:** Population characteristics

	Overall	Control group	Treated group
Mean age at time of birth	27.5	27.2	27.7
Mother’s educational attainment (%)			
Low (under 10 years)	40.8	43.3	38.7
Intermediate (10 to 12 years)	33.1	31.2	34.7
High (over 12 years)	22.1	21.4	22.8
Missing	4.0	4.1	3.8
Family educational attainment (%)			
Low (under 10 years)	21.4	22.6	20.5
Intermediate (10 to 12 years)	47.2	46.8	47.6
High (over 12 years)	29.9	29.1	30.5
Missing	1.5	1.6	1.5
Birthweight (%)			
Under 2500 g	94	93.7	94.2
Over or equal to 2500 g	5	5.1	4.9
Missing	1.1	1.2	0.9
Singleton (%)			
No	1.1	1.0	1.1
Yes	98.9	99.0	98.9
Mother partnered (%)			
No	15.4	15.1	15.7
Yes	84.6	84.9	84.3
Below mean income in the year before giving birth (%)	45.2	44.9	45.4
Employed at least 80% of the year before giving birth (%)	74.7	76.9	72.9
Psychiatric diagnosis in the 1–2 years before giving birth (%)	0.30	0.31	0.29
Eligible birth range	1 January 1981 to 31 December 1987	1 January 1981 to 25 March 1984	26 March 1984 to 31 December 1987
Sample size (births)	371 149	167 321	203 828

Income is reported only by calendar year. Some women may appear twice in these descriptive statistics if they gave birth multiple times during our tim eframe. The pre-reform characteristics are balanced across the treatment and control groups.

**Figure 2:**
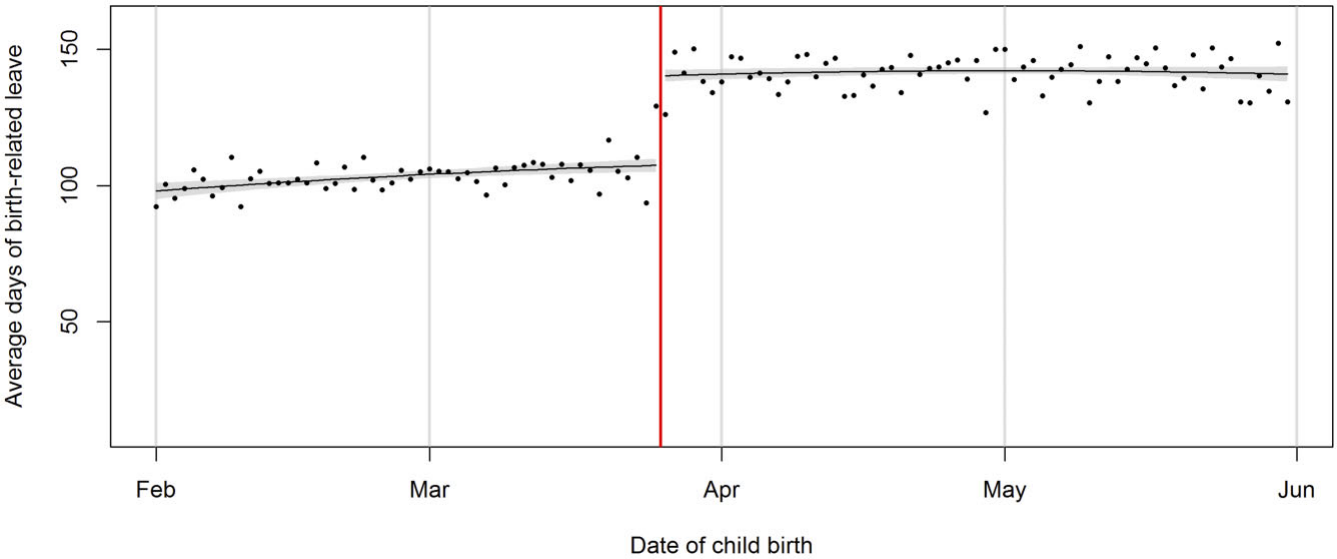
Average days of maternity leave taken by mothers before and after the reform. Each dot represents the mean number of days of birth-related leave taken for all children born on a specific day. The vertical line indicates 26 March 1984, the cut-off for eligibility for the reform

Eligibility for the reform was associated with an increase in parental leave duration of 32.85 days on average (95% CI: 29.20 to 36.49, *P *<* *0.001, Figure 2). For most births (82.2%) women took up additional leave, but those who did not had lower levels of education, were less likely to be in a partnership and more likely to have an income below average in the year preceding birth (Supplementary Appendix 4, available as Supplementary data at *IJE* online). There was no clear difference in the effect of the reform on leave duration by birthweight, income and family education (Supplementary Appendix 5, available as Supplementary data at *IJE* online). However, women who did not have a partner at the time of birth took limited additional leave (11.64, 95% CI: -1.37 to 24.65, *P *=* *0.08) compared with those who had a partner (35.57, 95% CI: 31.93 to 39.22, *P *<* *0.001).

**Figure 3:**
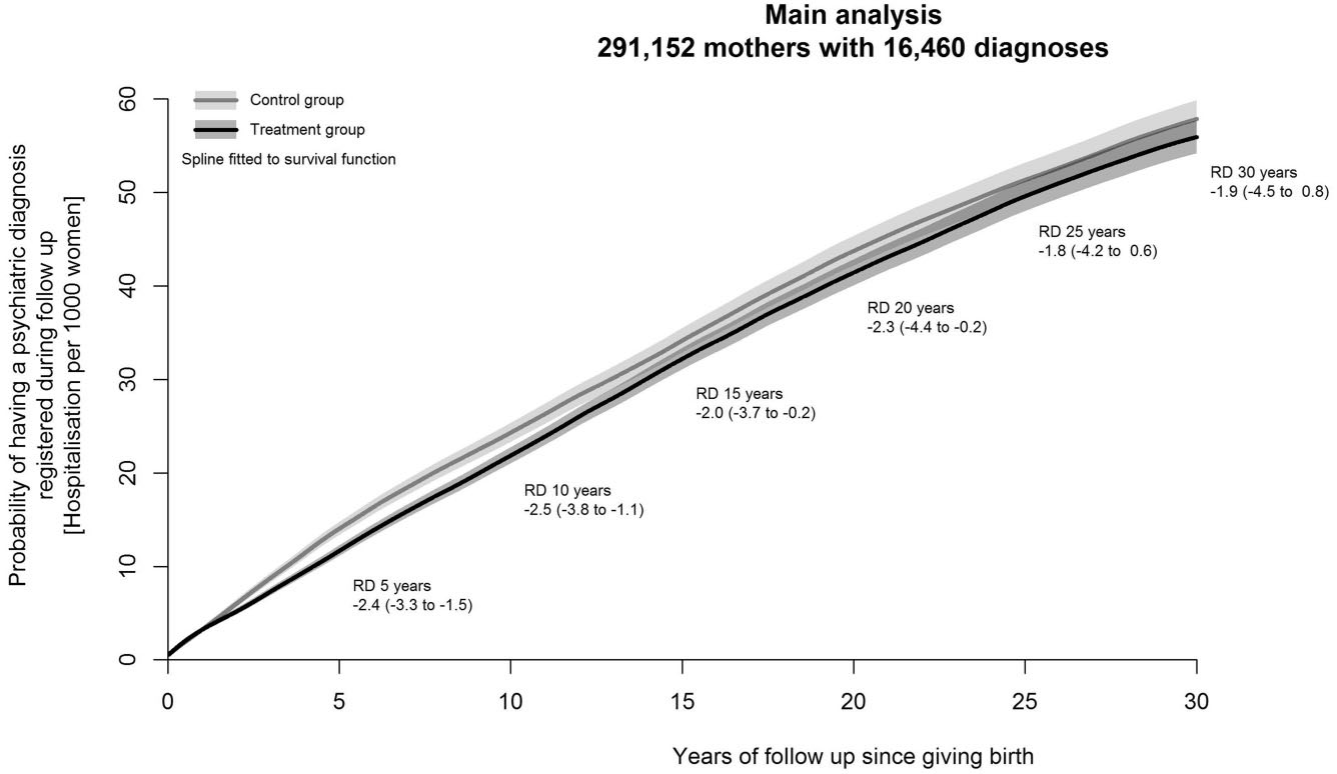
Cumulative incidence of psychiatric disorder diagnoses. RD refers to risk difference. Cumulative incidence curves are presented for the probability of psychiatric diagnostic during the 30-year follow-up. Risk is expressed per 1000 women

A corresponding discontinuity was observed in the risk of psychiatric diagnoses within 5 years of the birth, but was weaker when the full 30-year follow-up was considered (Supplementary Appendix 6, available as Supplementary data at *IJE* online). Women eligible for the reform had a lower probability of receiving a psychiatric diagnosis within 5 years (risk difference per 1000 women: −2.4, 95% CI: −3.3 to −1.5), 10 years (−2.5, 95% CI: −3.8 to −1.1), 15 years (−2.0, 95% CI: −3.7 to −0.2) and up to 20 years after the birth (−2.3, 95% CI: −4.4 to −0.2). The risk estimates are similar in magnitude at the 25-year (−1.8, 95% CI: −4.2 to 0.6) and 30-year follow-up (−1.9, 95% CI: −4.5 to 0.8; Figure 3).

**Figure 4:**
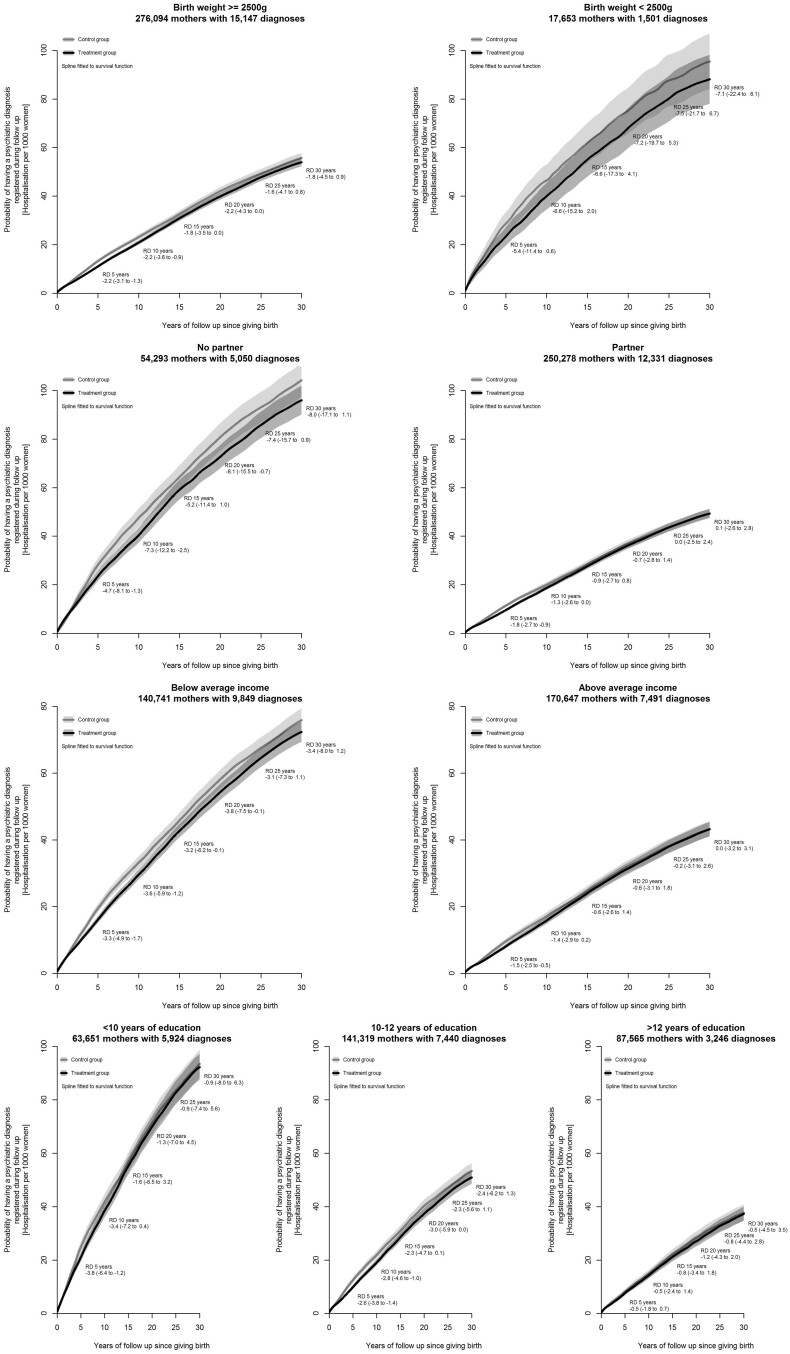
Cumulative incidence of psychiatric disorder diagnoses by subgroups of interest. RD refers to risk difference. Cumulative incidence curves are presented for the probability of psychiatric diagnostic during the 30-year follow-up. Risk is expressed per 1000 women

Turning to subgroups of interest (Figure 4), we found that the protective effect of the reform on mental health was concentrated among women with low education (−3.8, 95% CI: −6.4 to −1.2, test for different effect across subgroups: *P*-value = 0.03), who did not have a partner (−4.7, 95% CI: −8.1 to −1.3 at the 5-year follow-up, test for different effect across subgroups: *P*-value = 0.10) and who had below average income (−3.3, 95% CI: -4.9 to −1.7 at the 5-year follow-up, test for different effect across subgroups*: P*-value = 0.06).

We conducted several sensitivity analyses to check the robustness of our findings. First, adding a time-dependent variable to account for the transition from ICD-8 to 10 in 1994 did not substantially change our results (Supplementary Appendix 7, available as Supplementary data at *IJE* online). Second, we found no evidence of discontinuities in our outcome for ‘placebo reforms’ for years in which the reform did not take place (Supplementary Appendix 8, available as Supplementary data at *IJE* online). This increases our confidence that our results are not driven by secular trends, although other (unmeasured) confounders could still bias our results. Our estimates are also robust to different bandwidth sizes, ranging from 12 up to 45 months (Supplementary Appendix 9, available as Supplementary data at *IJE* online). Finally, we did not find a different pattern when restricting the analysis to the firstborn child (Supplementary Appendix 10, available as Supplementary data at *IJE* online).

## Discussion

In this quasi-experimental study, we found that women who gave birth after the introduction of the 1984 Danish reform took on average an additional month of parental leave. This increase in the duration of parental leave led to meaningful reductions in the risk of psychiatric diagnoses among the women giving birth during that period. We estimated that eligibility for the reform was associated with 2.4 fewer psychiatric diagnoses within 5 years per 1000 women giving birth, which corresponds to one psychiatric diagnostic potentially prevented for every 416 women eligible for the parental leave extension.

Three key findings warrant further discussion. First, the effects of the reform on the incidence of inpatient psychiatric diagnoses confirm the psychological benefits of parental leave.^18^^,^^19^ It is notable that we find such effects in a country like Denmark, which has traditionally been defined by generous and universal welfare benefits including child care.^31^ These results may also shed light on the association between maternal mental health and child outcomes.^32^ Increasing parental leave can be viewed as improving family resources and environment: mothers who are healthier may be able to invest more in their children. Improved maternal mental health may complement the increased time mothers can spend with newborns as a result of leave provisions, leading to better child outcomes.

Second, the use of registry data enabled us to document the impact of the policy on health trajectories up to several decades after the mother gave birth. Most of the existing literature to date has focused on short-term effects shortly after childbirth^7^ or at one time point later in life.^18^^,^^19^ Multiple channels could link the duration of parental leave to mental health in later life. A direct mechanism is relief from the stress associated with multiple work and family roles following birth. The perinatal period is associated with an increased risk of mental disorders, with adverse effects for the mother, child and family.^2^^,^^33–35^ Women experiencing mental health problems in the postpartum period are more likely to suffer from subsequent episodes of psychiatric disorders.^36^^,^^37^ Parental leave enables women to take time off work to recover from childbirth and bond with the new child. Parental leave policies may also have indirect effects with long-term implications: they increase employment, lifetime earnings and job continuity among mothers,^7^^,^^38^ which may in turn have positive effects on mental health beyond the immediate postpartum period. The reform assessed in this study has been associated with improvements in mothers’ income and career prospects,^24^ which might contribute to the positive mental health impacts we document up to 20 years after the reform. Our results highlight the importance of mid-life social policies on later life health among women. Early adulthood and the transition to parenthood are inflection points, when workplace exposures, family circumstances and public policies interact to shape opportunities that have long-term health consequences.^39^ Researchers have argued that the transition to parenthood is a critical window for determining both physical and mental health in mid-life and beyond, with existing disparities in mental health but also obesity and biomarkers of inflammation linked to risk factors stemming from the perinatal period.^40^ Our findings, that a substantial expansion of parental leave can reduce risks for poor mental health among working mothers up to 20 years after childbirth, suggest an important policy lever to support parents, relieve stress and influence health trajectories into older age.

Third, the protective effect of the reform was concentrated mainly among women with low education, lower than average income and no partner at the time of birth. These findings suggest that socioeconomically disadvantaged women, who are likely to struggle with the competing demands of work and care, may particularly benefit from an expansion of parental leave. The large effects on mental health in the first 5 to 10 years post-birth for these women suggests another important window for policy intervention, in particular because these women were also less likely to take up leave in the first place (only 12 additional days). Lowering barriers for at-risk subgroups of women might yield even larger effects.

Our study has several strengths. A major strength is that we relied on register-based data to obtain a large and unselected sample with little attrition. The study cohort contained all mothers who had given birth during our time frame, independent of their individual situation. This is an important improvement on the generalizability of existing studies which had mainly focused on first-time mothers. Second, we leveraged the exogenous sharp increase in leave duration induced by the reform to assess its causal effect on psychiatric diagnoses with fewer assumptions (e.g. exclusion restriction for instrumental variables) compared with other quasi-experimental designs.^30^^,^^41^

Our results are not without limitations. First, data on maternity leave duration were available only starting in January 1984. Similar to an intention-to-treat analysis in a randomized study, we consequently evaluated the impact of being eligible for the reform rather than the impact of taking-up the additional leave, which is likely to underestimate the impact of leave-taking on psychiatric diagnoses, due to non-compliance. Second, we had to focus our analysis on inpatient diagnoses to account for the transition from ICD-8 to ICD-10 in 1994. Inpatient psychiatric diagnoses arguably set a higher threshold to test the impact of a family policy on women’s mental health than outpatient diagnoses or self-reported symptoms. Our results likely underestimate the true effect of the expansion of parental leave on women’s mental health. We are probably only capturing the ‘tip of the iceberg’ by focusing on diagnosed psychiatric disorders, which are at the very end tail of the larger distribution of mental health problems which could have been affected by the reform. Based on other studies of the impact of parental leave reforms on mental health, we expect the effect on outpatient service use or self-reported depression to be protective.^19^^,^^42^ Third, the 1994 transition between disease classifications prevented us from looking at the impact of the reform on specific psychiatric diagnoses. Even after adjusting for time since the introduction of the ICD-10, we could not fully account for the large increase in anxiety and depression diagnoses after 1994. Finally, our analyses of long-term effects up to 30 years after the reform itself should be interpreted with caution. During that period, the Danish mental health care system underwent important changes in relation to the treatment of mental illness and the delivery of mental health services. We do not anticipate, however, that our eligible and ineligible groups would have been affected differently by these changes, and our placebo analyses provide further reassurance that we are picking up the effect of the reform and not of secular trends in health care delivery.

These findings are important in the current policy environment, with countries like France and the USA introducing or expanding considerably their parental leave provision. Prior to the 1984 reform, Danish parental leave was comparable in length to that of many states in the USA today, i.e. 12 weeks of (unpaid) leave for eligible workers. These results highlight the importance of studying indirect outcomes of these policies in addition to the immediate measures targeted by policy makers. Long-term maternal mental health effects of leave duration are a key spill-over effect of a policy which targets maternal employment and child development. These results add to the growing evidence base on the mental health consequences of social policies and their role in mitigating social inequalities in health.

## Ethics approval

These linkages were conducted as part of the DANLIFE cohort,^28^ which was approved by the Danish Data Protection Agency (record number 514–0262/18–3000).

## Supplementary Material

dyac198_Supplementary_Data

## Data Availability

Access to the DANLIFE cohort is available through collaborative agreements and granted access to the Danish registers by Statistics Denmark and the Danish Health Data Authorities. Please contact Professor Naja Hulvej Rod [nahuro@sund.ku.dk] for further information. Scripts to replicate our main results are available on [https://github.com/emiliecourtin/maternityleave].
