# Supplementary material for: The effect on women’s health of extending parental leave: a quasi-experimental registry-based cohort study
Source: Int J Epidemiol. 2022 Oct 14;52(4):993–1002. doi: 10.1093/ije/dyac198 (PMC10396408; doi:10.1093/ije/dyac198)
Supplement: dyac198_Supplementary_Data [file dyac198_supplementary_data.pdf]

**The effect of extending parental leave on women's mental health: a quasi-experimental registry-based cohort study**

**SUPPLEMENTARY FILE**

**Section 1**. The 1984 Danish Reform of Maternity Leave

Some form of support for maternity leave exists in Demark since 1901, although limited to women working in factories. In 1967, a reform was passed to ensure that all women would receive economic compensation during maternity leave if they were employed. A first large reform in 1980 gave women 14 weeks of maternity leave after birth and job protection, with income-dependent compensation. At the time of the 1984 reform, there was political and societal consensus about the importance of increasing birth-related leave to allow infants to spend more time with their parents. As a consequence, the policy went from proposal to effective stage within three months. The first proposal for the 1984 reform was put forward in October 1983 and the reform passed in December 1983.

The 1984 reform increased birth-related leave by almost 50%, from 14 to 20 weeks. The reform was composed of two steps, which added 6 and 10 weeks of shareable leave respectively on top of the existing 14 weeks of birth-related maternity leave. The first step applied to mothers who had given birth within 14 weeks of 1 July 1984. The second step applied to mothers who had given birth within 20 weeks of 1 July 1985. In our analyses, we focus on the first step of the reform only as potential mothers might have been able to anticipate the second policy change.

The additional 6 weeks were designed as parental leave, meaning that both mothers and fathers could potentially take that leave. In practice, very few households shared the leave: less than 3% of fathers used shareable leave in 1984 when that option became available.(1) The benefit level was high, at 90% of previous pay. The reform was universal: all potential recipients receive the same entitlement based on the date of childbirth.

The reform was implemented on 1 July 1984. This official start date was accompanied by a period of transition. Mothers who were already on birth-related leave under the previous policy at the start date were automatically eligible for the expanded leave. As noted by Rasmussen, the cut-off date for the reform was in practice 12 weeks and 6 days before 1 July 1 1984 (2). We consequently use as a cut-off for eligibility to the reform 26 March 26 1984.

A potential concern with the measurement of the health effects of social policies is that other changes relevant for health might co-occur. As suggested by Matthay and colleagues(3), we take advantage in this study of a feature of the policy that is not likely to be relevant for other social policies. Specifically, we use an identification strategy that focuses on the threshold for eligibility to the reform. We do not leverage changes across Danish regions or overtime using a difference-in-differences analysis but use the policy rule that defined eligibility to the reform. That approach is less vulnerable to bias from co-occurring social policies for two reasons. First, we use a very precise threshold based on the exact date of childbirth, which is highly unlikely to be shared with another reform in another policy domain (children born after 23 March 1984). Second, other reforms might have been implemented after March 1984, with an impact on women's mental health. This would bias our findings if we had a reason to believe that the treated and control groups have been exposed to different reforms over time. It is not the case here.

**Section 2**. McCrary test of manipulation of the assignment variable

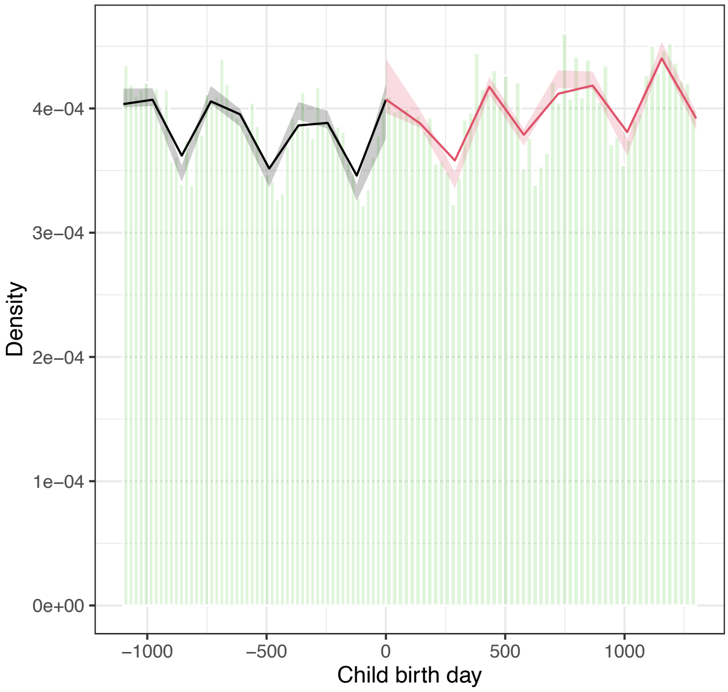


*Footnote:* This figures presents the number of births relative to the cut-off for eligibility to the reform (not eligible in black and eligible in red) for up to 1200 days on each side of the cut-off. 0 represents the cut-off for eligibility.

**Section 3.** Technical Appendix

*Regression Discontinuity Design*

To assess the effect of the extension of maternity leave on maternal mental health, we implemented a regression discontinuity design (RDD). The reform increased the duration of paid maternity leave for all births occurring on or after 26 March 1984. We estimated the effect of eligibility to the treatment (parents' entitlement to an additional 6 weeks of leave) by comparing the outcomes of interest (leave duration or time to psychiatric diagnosis) of women who benefitted from reform with those who did not. Mothers in our study are allocated to either a control group (which did not benefit from the extended leave) or to a treated group (which was eligible to an additional 6 weeks of leave) based on the child's date of birth. Because the reform was announced less than nine months before the eligibility cut-off, assignment to the treatment is deemed as good as random, mimicking a randomised control trial. This was confirmed by a McCrary test of manipulation of the assignment variable (supplementary section 2).

We first estimated the impact of eligibility the reform on parental leave duration using a sub-sample of mothers for whom we had information on leave-taking. This information is available starting in January 1984 and we consequently include women who gave birth approximately two months before and after the reform (1 February 1984 to 31 May 1984, n=17,719). We model the change in parental leave duration by the following reduced form model:

$$duration_{i,b,t} = \beta_0 + \beta_1 D_{i,b} + f(R_{i,b}) + \eta_{i,b,t} \quad (1)$$

Where $duration_{i,b,t}$ is the family leave duration for individual *i* at time *t* giving birth at time *b*. $D_{i,b}$ is a binary variable taking the value of 1 if the mother gave birth up to 2 months after the cut-off date (the treated group), and of 0 if the mother gave birth up to 2 months after to the cut-off date (the control group). $R_{i,b}$ is a measure of the distance between the child's date of birth (measured in days) and the cut-off for eligibility to the reform (26 March 1984) modelled as linear and squared.

Second, we estimated the effect of eligibility to the reform on time to psychiatric diagnosis. We considered a much larger sample of women for these analyses and included women who gave birth between 1 January 1981 and 31 December 1987 (n=291,143 mothers after removing 933 mothers whose date of birth was not available). We implemented a Cox model to account for survival data, parameterizing the cohort effect as linear and squared and with reform groups as strata and time in the study since birth as the underlying variable.(4) We model the hazard of first diagnosis by the following reduced form model:

$$h_{i,b,t} = h_{0,t} \exp\left( \propto_0 + \propto_1 D_{i,b} + f(R_{i,b}) + \eta_{i,b,t} \right) \quad (2)$$

Where $h_{i,b,t}$ is the hazard of the first diagnosis at time *t* for individual *i* giving birth at time *b*. $D_{i,b}$ is a binary variable, indicating whether individual *i* gave birth at a time *b* eligible for extended maternity leave. It takes the value of 1 if the mother gave birth up to 39 months after the cut-off date (the treated group), and of 0 if the mother gave birth up to 39 months after the cut-off date (the control group). $R_{i,b}$ is a measure of the distance between the child's date of birth (measured in days) and the cut-off for eligibility to the reform (26 March 1984). We included this variable as linear and squared terms.

We then predicted cumulative incidence functions adjusted for the cohort effect and ICD version regime for each strata and estimated the risk and associated confidence intervals for each groups in 5-year intervals for the full follow-up period. We reported the absolute risk difference between the groups and associated 95% confidence intervals.

*Interpretation of the estimates*

The estimates we obtained from these RDD models are interpreted as intent-to-treat parameters. We estimate the effect of *eligibility* to the reform on maternity leave duration and time to psychiatric disorder diagnosis. Eligibility was a deterministic function of birthdate, which makes our approach a sharp regression discontinuity design. A small proportion of women who were eligible did not take up the additional leave (17.8%). As we do not have leave taking information for our full sample, we were unable to estimate the average effect of six additional weeks of leave on time to psychiatric diagnosis (*i.e.* the impact of the reform on those mothers who actually took up the additional leave offered by the reform) using a two-stage residual inclusion approach to account for survival data.(5)

*Effect modification*

To investigate whether there were differential effects across subgroups, we stratified our analyses by child birth weight, partnership status, family education level and income. Low birth weight was coded as below 2500g at birth *vs.* over or equal to 2500g). Mother's partnership status at the time of birth was coded as partnered *vs.* not partnered based on information on the 1st of January of the year of birth. Family educational attainment was coded as under 10 years of education, 10 to 12 years and above 12 years with the family assigned to the highest educational level registered for either parent. Income was coded as above *vs.* below the predicted average income).

We stratified our analyses by these sub-groups of interest and report plots displaying (1) the effect of eligibility to the reform on leave duration for each of the subgroups and (2) the effect of eligibility to the reform on the probability of receiving a psychiatric diagnosis for our full follow-up period for each of these categories.

*Sensitivity analyses*

We conducted four main sensitivity analyses. First, we added a time-dependent variable to account for the transition from ICD-8 to 10 in 1994. Second, we tested the effect of 'placebo' reforms for years in which the reform did not take place. We considered the following cut-offs, with a 15-month bandwidth on each side:

- 26 March 1982 (placebo reform)
- 26 March 1984 (true reform)
- 26 March 1986 (placebo reform)

*Definition of the placebo reforms in our sample*

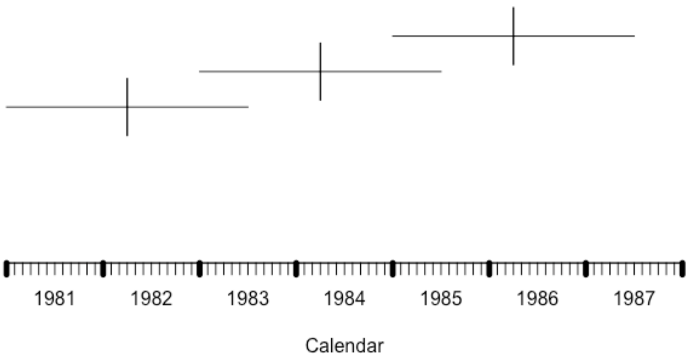

We report the effect of these placebo reforms both at the five-year follow-up as well as for the full duration of follow-up. Third, we tested whether our estimates we robust to different bandwidth sizes, ranging from 12 to up to 45 months. Finally, we assess whether focusing on the firstborn child only affects our results. In the models on psychiatric diagnoses, we clustered standard errors at the mother's level to account for mothers potentially contributing multiple children (repeated births) to our model. To further assess whether this approach impacted our estimates, we re-ran our models on the sub-sample of women who gave birth to their first child in our time window.

**Section 4.** Characteristic of mothers by leave take-up

| | No additional leave | Additional leave | Total |
|---|---|---|---|
| Age at birth | 26.7 | 27.8 | 27.6 |
| Low education | 51.4% | 36.9% | 39.4% |
| Medium education | 20.6% | 36.2% | 33.4% |
| High education | 15.2% | 25% | 23.3% |
| *Missing on education* | *12.8%* | *1.9%* | *3.9%* |
| Low education (family) | 36.2% | 15.9% | 19.5% |
| Medium education (family) | 33.2% | 51% | 47.9% |
| High education (family) | 24.3% | 32.6% | 31.1% |
| *Missing on education (family)* | *6.2%* | *0.5%* | *1.5%* |
| Normal birth weight (>=2500g) | 90.1% | 95.8% | 94.8% |
| Low birth weight (<2500g) | 6.1% | 3.9% | 4.3% |
| *Missing on birth weight* | *3.8%* | *0.2%* | *0.9%* |
| Singleton | 99.1% | 98.7% | 98.8% |
| Below mean income in the year before giving birth | 85.5% | 34.9% | 43.6% |
| Employed 80% the year before giving birth | 71.3% | 72.2% | 72.1% |
| Not in a partnership | 27.2% | 9.1% | 12.3% |
| Sample size (births) | 1799 | 8303 | 10102 |

*Notes*: Leave take-up was defined as taking at least 99 days of leave post reform.

**Section 5.** Average days of maternity leave taken by mothers before and after the reform, by subgroups of interest

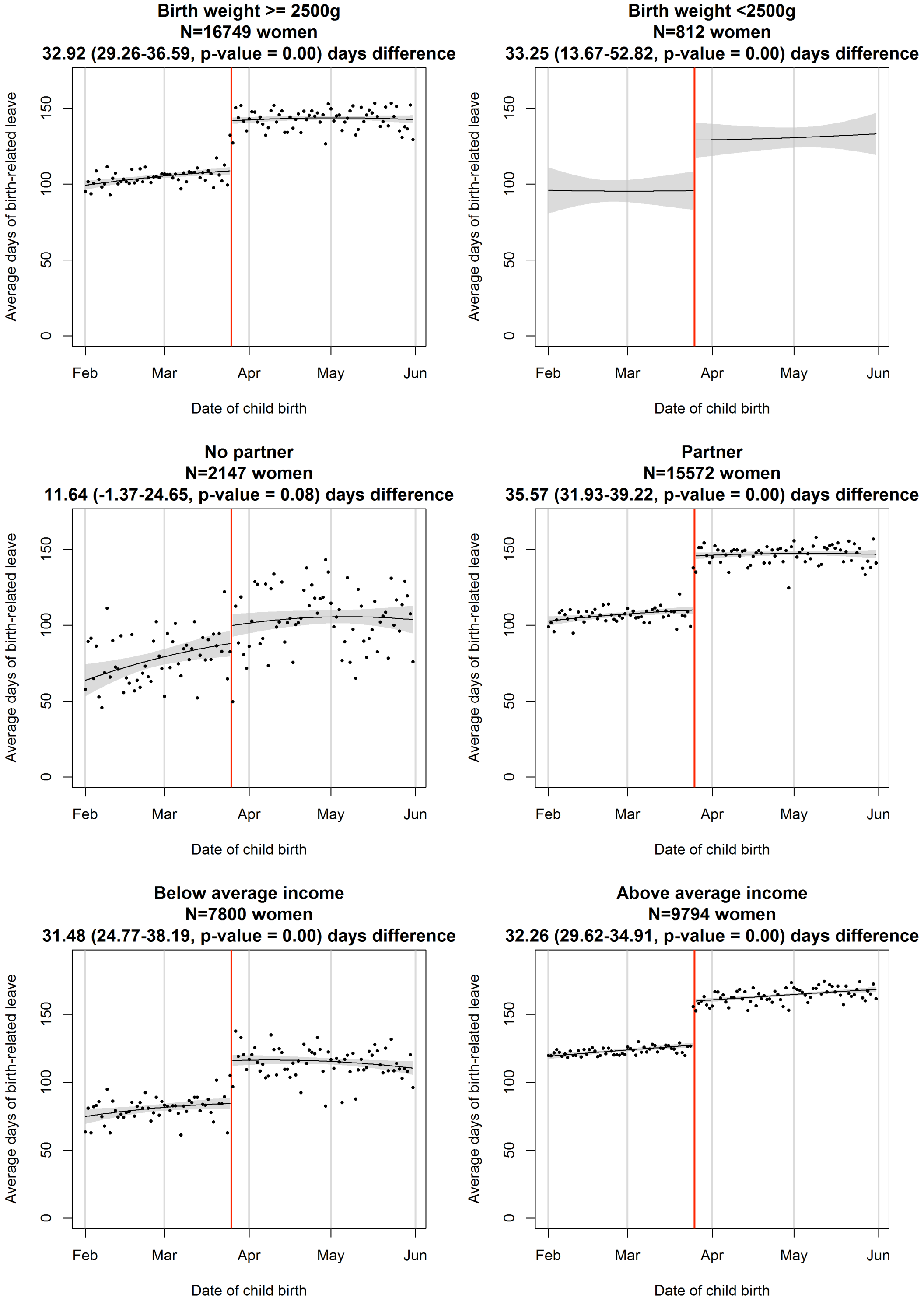

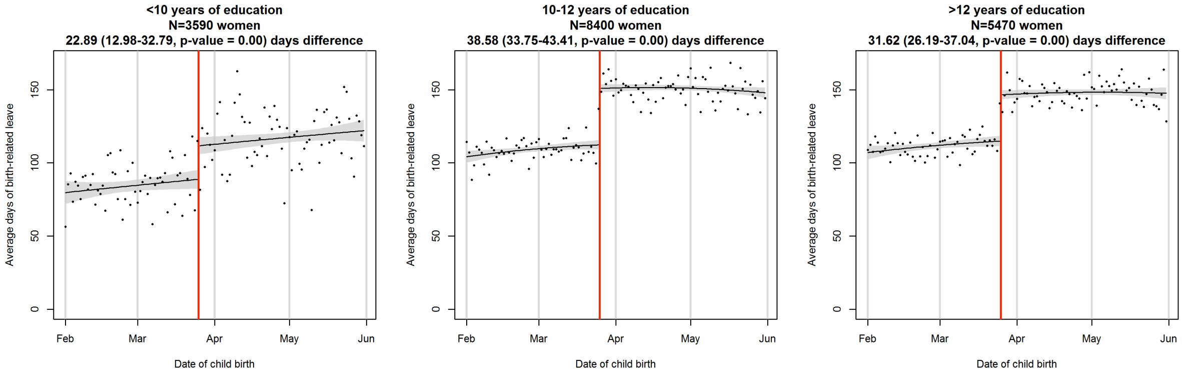


*Footnote*: Each dot represents the mean number of days of birth-related leave taken for all children born on a specific day. Given the small sample sizes for low birth weight births, these dots are not reported for data protection The vertical red line indicates 26 March 1984, the cut-off for eligibility to the reform.

**Section 6.** Rate of psychiatric diagnoses per 1000 person-years at 5-years and 30-years of follow-up

*Follow up from 0 to 5 years*

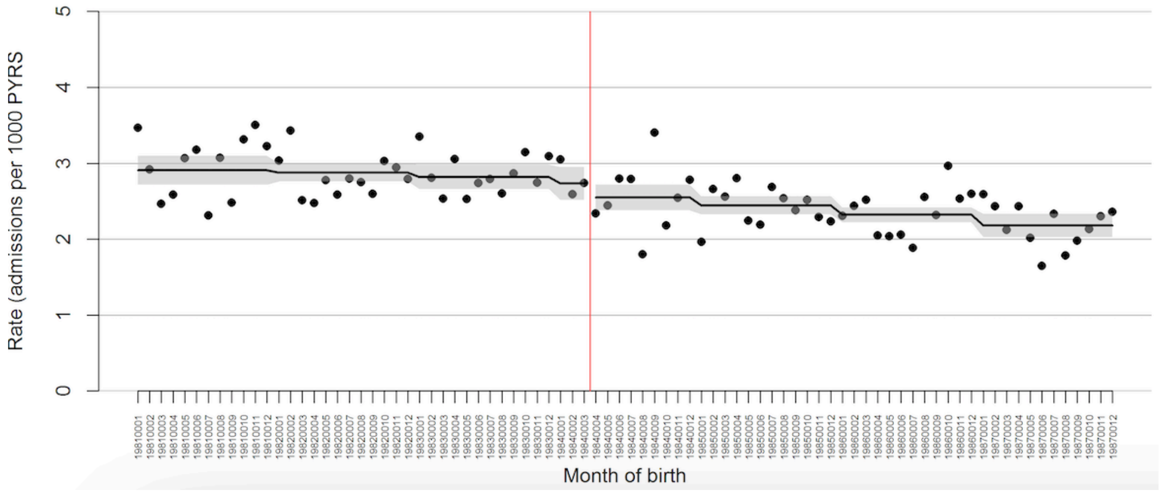


*Follow up from 0 to 30 years*

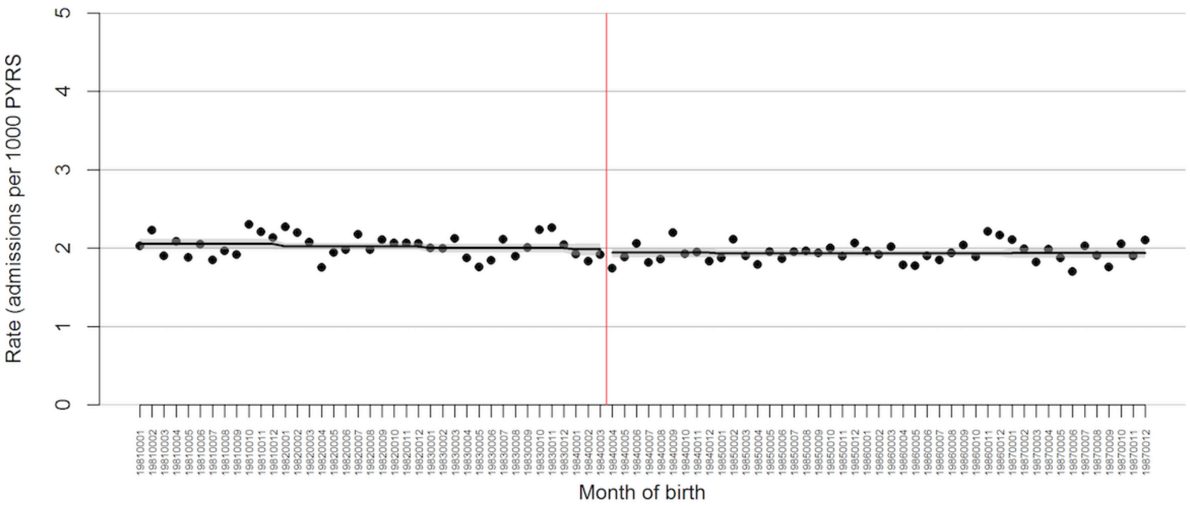


*Footnote*: Each dot represents the rate of maternal inpatient diagnoses per 1,000 person-years for children born on a specific day. The vertical line indicates 26 March 1984, the cut-off for eligibility to the reform.

**Section 7.** Cumulative incidence of psychiatric disorder accounting for the transition from ICD-8 to 10 in 1994

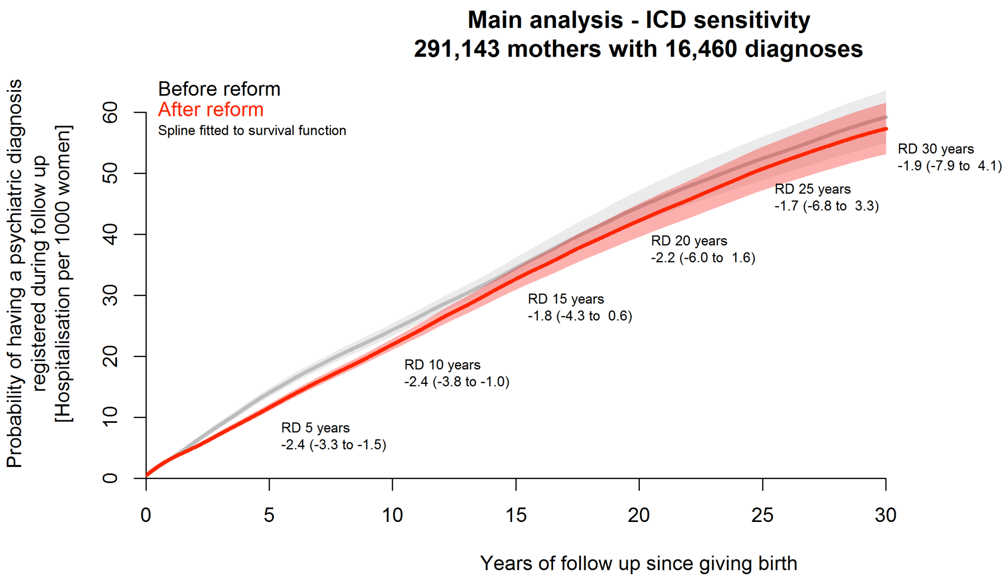


*Footnote*: RD refers to risk difference.

**Section 8.** Placebo reform tests

*Risk difference at 5-year follow-up (placebo vs. true reform)*

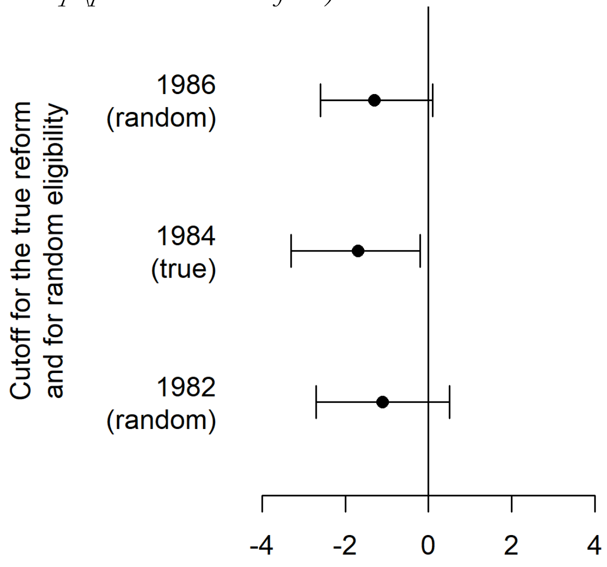

*Risk difference for the full follow-up period (placebo vs. true reform)*

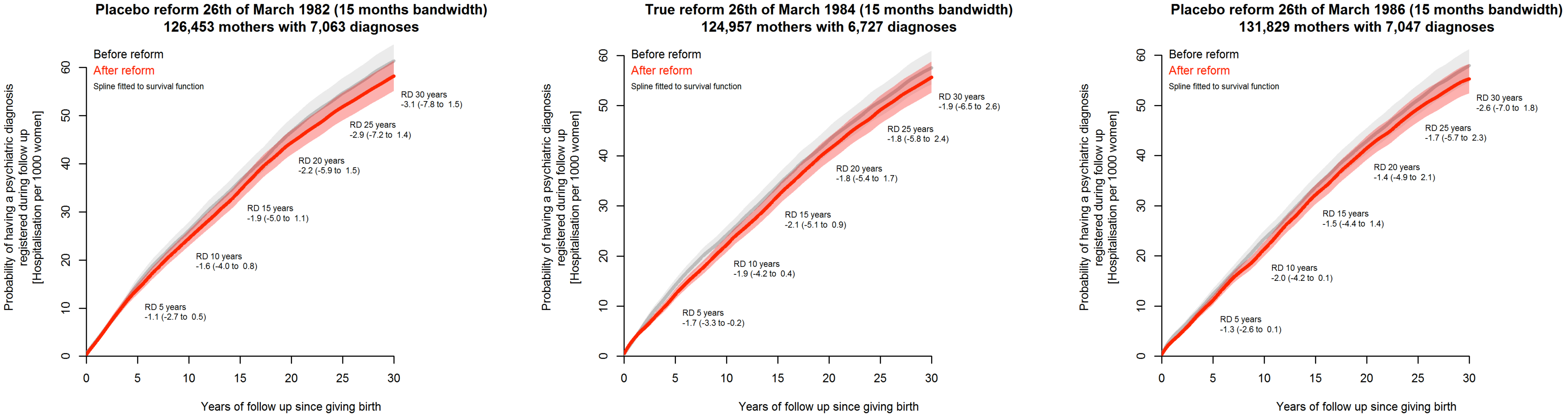


*Footnote*: RD refers to risk difference

**Section 9.** Risk difference between the treatment and control group per 1000 women at 5-year follow-up with different bandwidth sizes

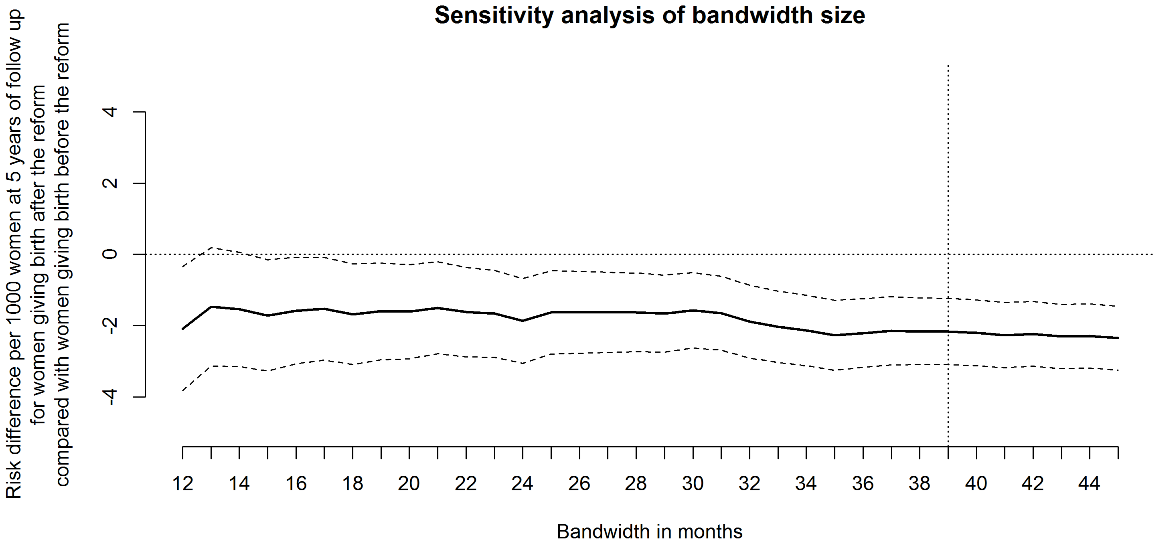


*Footnote*: The bandwidth is displayed in months and ranges between 12 and 45 months. The vertical line displays the bandwidth chosen for our main analyses (39 months).

**Section 10**. Cumulative incidence of psychiatric disorder diagnoses by parity order

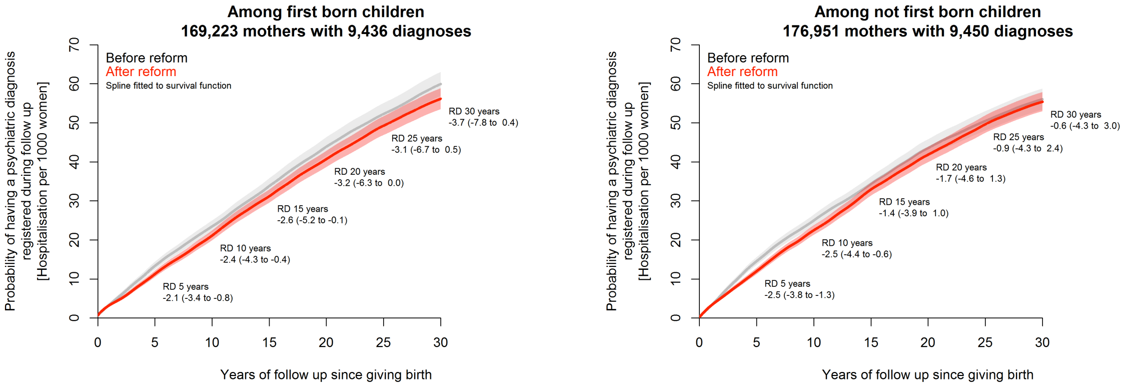


*Footnote*: RD refers to risk difference

**References**


1. To L. The signaling role of parental leave. 2018.
2. Rasmussen AW. Increasing the length of parents' birth-related leave: The effect on children's long-term educational outcomes. Labour Economics. 2010;17(1):91-100.
3. Matthay EC, Gottlieb LM, Rehkopf D, Tan ML, Vlahov D, Glymour MM. What to Do When Everything Happens at Once: Analytic Approaches to Estimate the Health Effects of Co-Occurring Social Policies. Epidemiologic Reviews. 2022;43(1):33-47.
4. Cox DR. Regression Models and Life-Tables. Journal of the Royal Statistical Society: Series B (Methodological). 1972;34(2):187-202.
5. Tchetgen Tchetgen EJ, Walter S, Vansteelandt S, Martinussen T, Glymour M. Instrumental variable estimation in a survival context. Epidemiology. 2015;26(3):402-10.
